# Investigating the Effects and Potential Mechanisms of Astragalus Root Against Diabetic Nephropathy Based on Bioinformatics Analysis and In Vitro Validation

**DOI:** 10.3390/ijms27104641

**Published:** 2026-05-21

**Authors:** Jie Li, Subinur Ahmattohti, Ying Gao, Xiangqin Xie, Jasur Kasim, Liang Feng, Baojian Li, Shuliang Niu, Jianguang Li

**Affiliations:** 1 School of Pharmacy, Xinjiang Second Medical College, Karamay 834000, China; lijie@xjsmc.edu.cn (J.L.); 18016910278@163.com (S.A.); kyc@xjsmc.edu.cn (X.X.); jasur2025@163.com (J.K.); 2 Science and Innovation Center of Xinjiang Second Medical College, Karamay 834000, China; 18829190996@163.com (Y.G.); libaojian@xjsmc.edu.cn (B.L.); 3 Shaanxi Collaborative Innovation Center of Chinese Medicine Resources Industrialization, Shaanxi University of Chinese Medicine, Xianyang 712046, China; 4 School of Basic Medical Sciences, Xinjiang Second Medical College, Karamay 834000, China; fengliang@xjsmc.edu.cn

**Keywords:** Astragalus root, kaempferol, diabetic nephropathy, network pharmacology, molecular docking, molecular dynamics simulation

## Abstract

Astragalus root, a traditional Chinese herbal remedy, has shown potential benefits against diabetic nephropathy (DN). However, the mechanisms driving its effects remain poorly understood. This study explored the molecular pathways through which Astragalus root improves DN. To identify possible targets and mechanisms of Astragalus root in DN treatment, we applied network pharmacology, molecular docking, molecular dynamics simulation, and in vitro assays. Network pharmacology screening uncovered 46 overlapping targets between Astragalus root and DN. Protein–protein interaction (PPI) network analysis identified five core candidate targets: CASP3, VEGFA, CTNNB1, MYC, and PRKCB (PKCβ). KEGG pathway analysis indicated that the AGE-RAGE signaling pathway was the most significantly enriched. Molecular docking revealed that quercetin, β-carotene, daidzein, capsaicin, and kaempferol—potential bioactive components of Astragalus root—bound strongly to each of the five core targets. Molecular dynamics simulations further confirmed the conformational stability of kaempferol when complexed with these target proteins. In vitro experiments showed that kaempferol markedly reduced protein levels of α-SMA, Col I, and Col IV; lowered secretion of TNF-α, IL-6, and IL-1β; and decreased ROS and MDA content. Additionally, kaempferol’s therapeutic effects were mediated through suppression of the AGE-RAGE-PKCβ-TGF-β1 signaling axis. This work identified kaempferol, a bioactive ingredient of Astragalus root, as a potential therapeutic agent against DN, along with its target pathways. These findings provide a scientific foundation for its clinical translation.

## 1. Introduction

Diabetic nephropathy (DN) is a major driver of chronic kidney disease and end-stage renal failure, standing as one of the most frequent microvascular consequences of diabetes [1]. According to International Diabetes Federation data, 537 million adults worldwide had diabetes in 2021, a number that is expected to climb to 788 million by 2045, with approximately 40% of diabetic individuals projected to develop DN over their lifetimes [2]. DN is pathologically defined by glomerular basement membrane thickening, mesangial cell injury, and glomerulosclerosis [3]. Without early intervention, these changes lead to severe clinical outcomes. Accumulating evidence indicates that DN pathogenesis involves complex interactions among multiple factors, including disordered glucolipid metabolism, oxidative stress, inflammation, and renal fibrosis [4]. The Guidelines for the Integrated Traditional Chinese and Western Medicine Prevention and Treatment of Diabetic Kidney Disease (2025) note that current DN management primarily targets hyperglycemia, hypertension, proteinuria, and hyperuricemia [5]. Although ACEI/ARBs and SGLT2 inhibitors can slow DN progression to some degree, their effects largely focus on early risk factor control and end-stage renal replacement therapy. These drugs offer limited benefit in halting disease advancement and often carry adverse effects [6]. Therefore, discovering new therapeutic strategies with multi-target and systemic regulatory capabilities is critical for delaying DN progression and improving patient outcomes.

Traditional Chinese Medicine (TCM) is a well-recognized complementary and alternative medical system that plays an important role in chronic kidney disease care. Its core philosophy for treating complex chronic conditions derives from classical theories, focusing on restoring Yin–Yang balance, regulating Qi and blood circulation, and harmonizing organ functions. In clinical practice, TCM uses multi-component, multi-target herbs to personalize patient management, thereby counteracting pathological processes such as inflammation, oxidative injury, and fibrosis. This approach aligns well with DN’s complex pathophysiology, positioning TCM as a promising adjunct to conventional therapy [7]. Various herbal extracts and their active ingredients have been reported to improve renal function, regulate immunity, and reduce proteinuria and blood glucose in DN [8,9,10]. Nonetheless, the precise mchanisms and clinical value of these effects need further clarification.

Astragalus root (*Astragalus membranaceus* (Fisch.) Bge. var. *mongholicus* (Bge.) Hsiao) is a classic Qi-tonifying herb (Qi denotes vital energy in TCM) known for replenishing Qi. It has been shown to reduce proteinuria and lower blood glucose in various herbal formulas. With the advantages of sustained efficacy, a favorable safety profile, and low cost, this herb is widely used in clinical practice. Further clinical evidence indicates that Astragalus, used either as a monotherapy or adjunctively with Western drugs, markedly decreases urinary protein excretion, enhances renal function, and retards disease progression in individuals with DN [11]. In addition, basic research has shown that Astragalus and its active components exert effects through multiple mechanisms. For instance, total flavonoids of Astragalus root reduce renal fibrosis and enhance filtration barrier function by suppressing inflammation and oxidative stress, thereby slowing DN [12]. Astragalus polysaccharides have also been shown to improve renal function in DN by promoting autophagy and inhibiting apoptosis and inflammation in kidney tissues [13]. Astragaloside I (ASI) exerts protective effects against early diabetic nephropathy through antioxidant activity, reduction in extracellular matrix (ECM) accumulation, and regulation of the cell cycle [14]. These findings demonstrate the considerable efficacy of Astragalus root—derived active components—which tonifies Qi, uplifts Yang, strengthens defensive Wei Qi, and consolidates the exterior–in treatment of DN. However, the detailed mechanisms—particularly the specific active ingredients, targets, and signaling pathways involved—remain to be fully defined.

Network pharmacology offers a fresh perspective for understanding TCM theory and has become a key tool for unraveling the “multi-component, multi-target” synergy of herbal medicines, especially in complex diseases like metabolic disorders [15]. Building on this, integrating molecular docking and molecular dynamics simulation enables validation of interaction patterns and dynamic stability between active ingredients and core targets at the molecular level, thus supporting the screening and confirmation of network-based predictions [16]. Currently, the research paradigm that integrates bioinformatics approaches with in vitro experimental validation has emerged as a pivotal breakthrough in the field of Traditional Chinese Medicine for the prevention and treatment of metabolic diseases [17]. In this study, we adopted a comprehensive approach combining network pharmacology, molecular docking, molecular dynamics simulation, and in vitro validation to systematically explore the therapeutic potential of Astragalus root against DN. This approach aims to identify the key active components and potential targets of Astragalus, and to elucidate its regulatory mechanisms through in vitro experiments, thereby advancing the understanding of its therapeutic prospects.

## 2. Results

### 2.1. Screening of Potential Targets and Pathways of Astragalus in the Treatment of DN Based on Network Pharmacology

**Figure 1:**
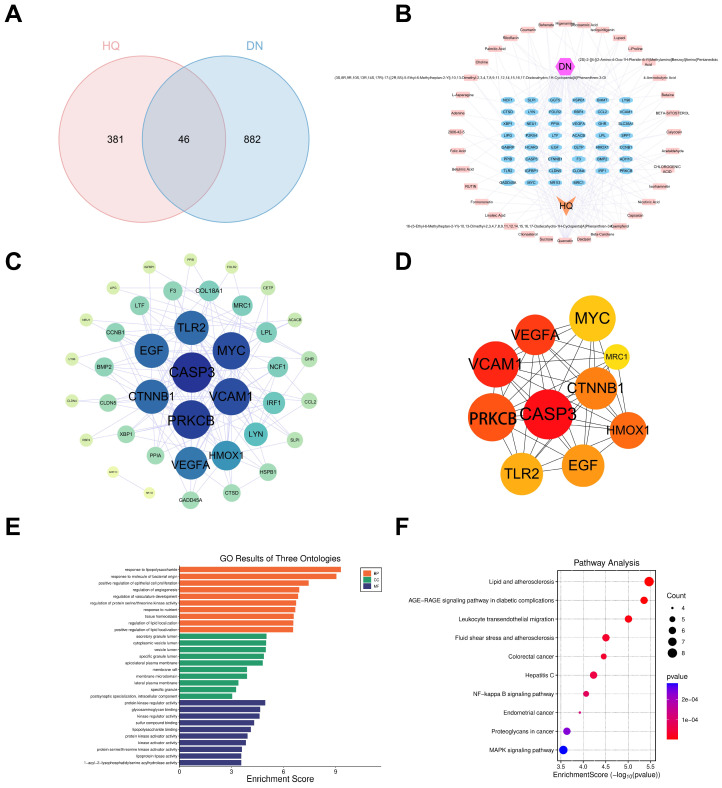
Potential targets and pathways of Astragalus membranaceus in the treatment of DN based on network pharmacology. (**A**) Venn diagram of active components of Astragalus membranaceus and disease targets of DN; (**B**) network diagram of herb-component–target-disease; (**C**) PPI network diagram of intersection targets; (**D**) 10 hub genes screened by CytoHubba plugin centrality analysis; (**E**) GO functional enrichment analysis; (**F**) KEGG pathway enrichment analysis.

From the four databases, we obtained 928 DN-related targets after duplicate removal. Using TCMSP with OB ≥ 30% and DL ≥ 0.18, we identified multiple active components of Astragalus root. Corresponding targets were predicted via SwissTargetPrediction, yielding 427 targets after deduplication. The intersection of Astragalus targets and DN targets produced 46 potential therapeutic targets (Figure 1A). To visually illustrate the interactions between the active components of Astragalus and DN-related targets, a herb-component–target-disease network was constructed (Figure 1B). The 46 intersecting targets were submitted to STRING to construct a PPI network (confidence threshold 0.40). Visualization with Cytoscape revealed a network of 40 nodes and 143 edges (Figure 1C). Centrality analysis using the MCC algorithm in CytoHubba identified 10 hub genes, including CASP3, VEGFA, CTNNB1, MYC, and PRKCB (Figure 1D), along with five potential components: quercetin, β-carotene, daidzein, capsaicin, and kaempferol. These potential genes and components likely play central roles in Astragalus root’s therapeutic action against DN.

GO analysis of the 46 intersecting targets showed broad involvement in 4031 biological pathways, covering 1323 GO terms and 77 KEGG pathways. GO annotations were subdivided into 1140 BP terms, 79 CC terms, and 104 MF terms. BP terms were notably enriched in response to lipopolysaccharide, response to bacterial-origin molecules, and positive regulation of epithelial cell proliferation. CC analysis highlighted localization in secretory granule lumen, cytoplasmic vesicle lumen, and vesicle lumen. MF analysis showed enrichment in protein serine/threonine kinase activity, kinase binding, and DNA-binding transcription factor binding (Figure 1E). KEGG analysis revealed significant enrichment of the AGE-RAGE signaling pathway and MAPK signaling pathway (Figure 1F).

### 2.2. Molecular Docking Validation

**Figure 2:**
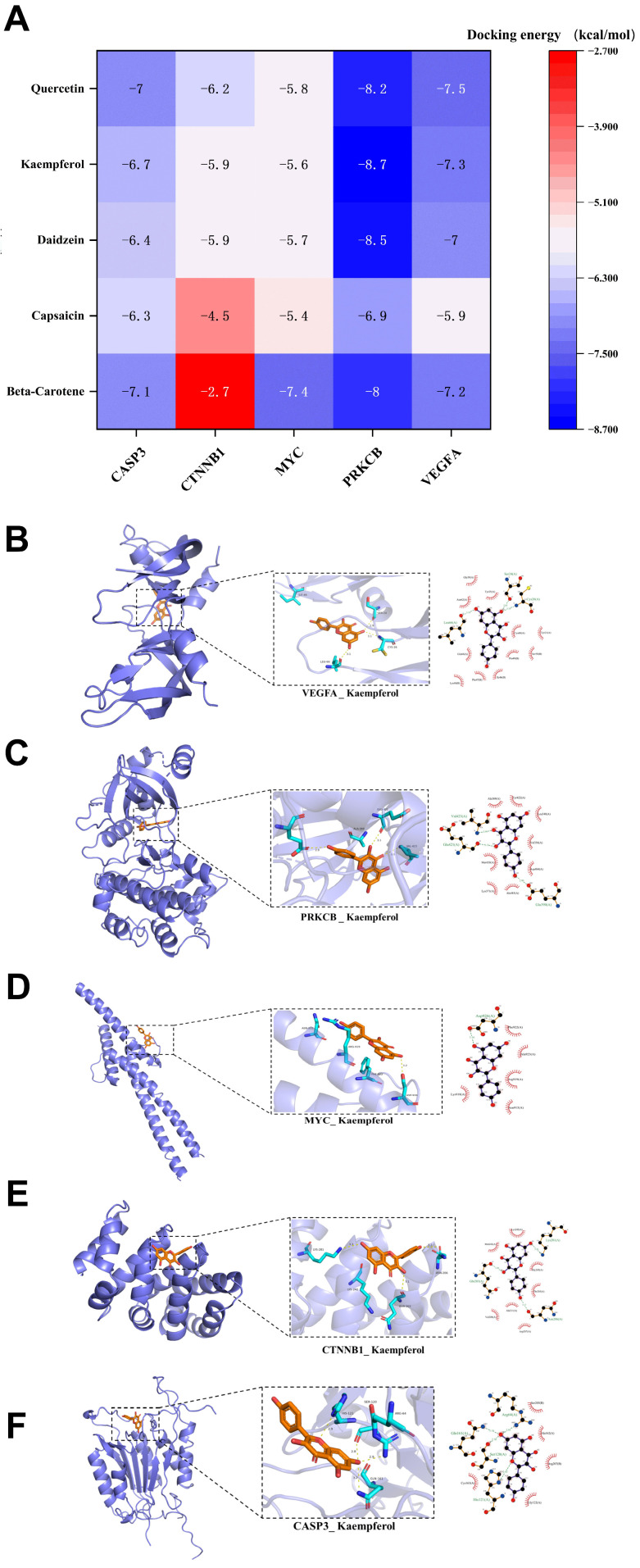
Molecular docking map of the potential components of Astragalus and core targets. (**A**) Heatmap of docking score between potential components and target sites. (**B**–**F**) Molecular docking of the structure for kaempferol with VEGFA, PRKCB, MY, CTNNB1, CASP3.

To validate binding between the five potential Astragalus components and the prioritized hub targets (VEGFA, PRKCB, MYC, CTNNB1, CASP3), we performed molecular docking with AutoDock. Results showed stable binding affinities between Astragalus components and all target proteins (Figure 2A). We calculated docking score, with daidzein, kaempferol, and quercetin showing docking scores below −5 kcal/mol. Kaempferol exhibited the lowest overall docking score and was selected for further visualization (Figure 2B–F).

Detailed structural analysis revealed key molecular recognition mechanisms: kaempferol formed stable complexes through conventional hydrogen bonds, carbon–hydrogen bonds, and hydrophobic interactions dominated by π-alkyl, π-anion, π-cation, π-sigma, π-sulfur, and π-lone pair contacts (e.g., with valine, alanine, and leucine hydrophobic pockets), as well as π-π stacking. These specific intermolecular forces stabilized the ligand–protein complexes and provided mechanistic insight into Astragalus root’s regulation of DN-related signaling nodes.

### 2.3. Molecular Dynamics Simulation

**Figure 3:**
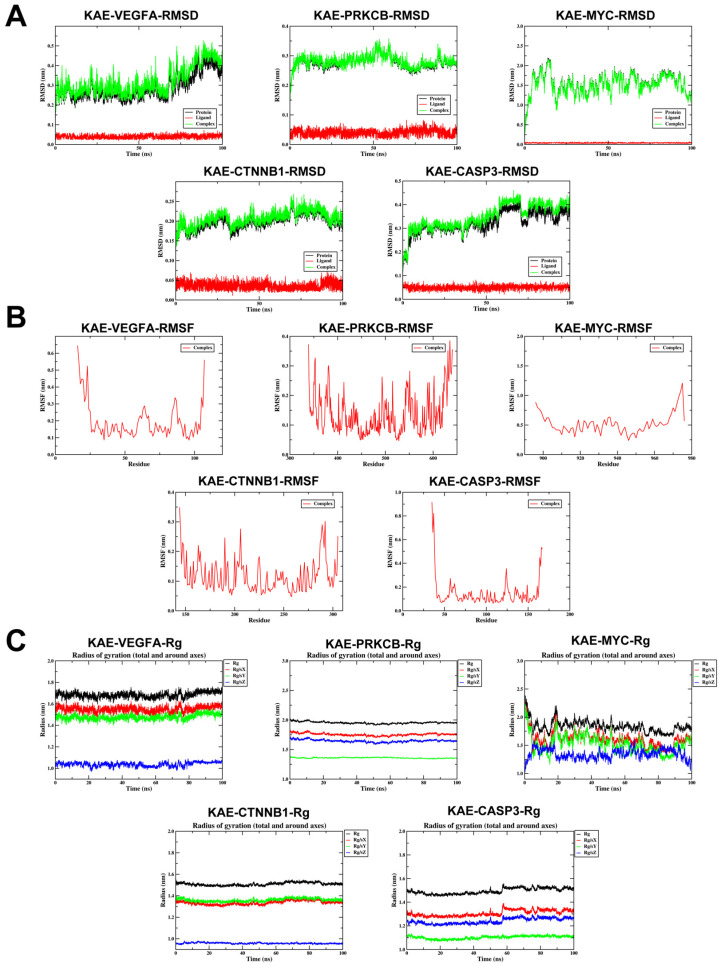

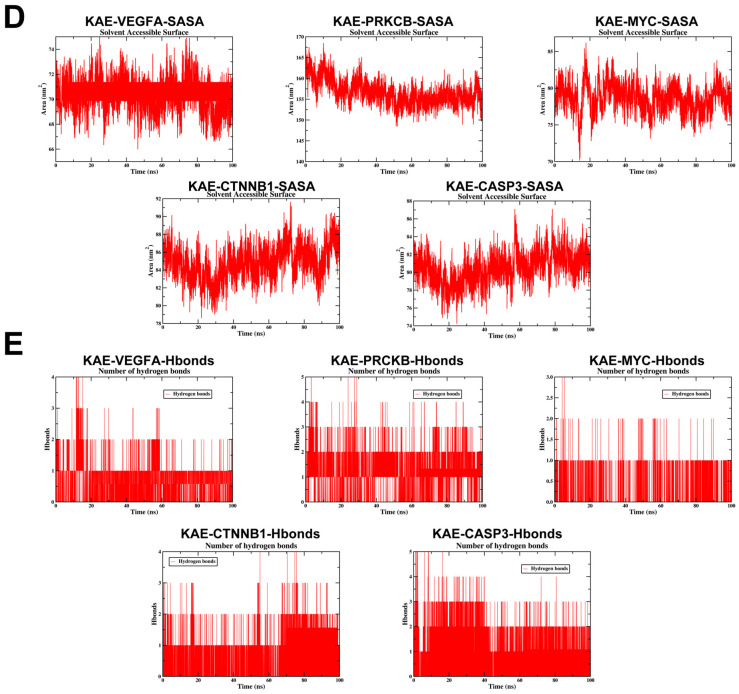
Molecular dynamics simulation stability analysis of kaempferol and core target complex. (**A**) The RMSD values for each target protein–kaempferol complex. (**B**) Variations in protein flexibility throughout the kaempferol simulation. (**C**) Rg rate curve of the protein–kaempferol complex. (**D**) SASA of the protein–kaempferol complex during 100 ns simulation. (**E**) Dynamics of hydrogen bonding as observed in the molecular dynamics simulations. (Note: RMSD: root-mean-square deviation; RMSF: root-mean-square fluctuation; SASA: solvent accessible surface area; Rg: radius of gyration).

**Figure 4:**
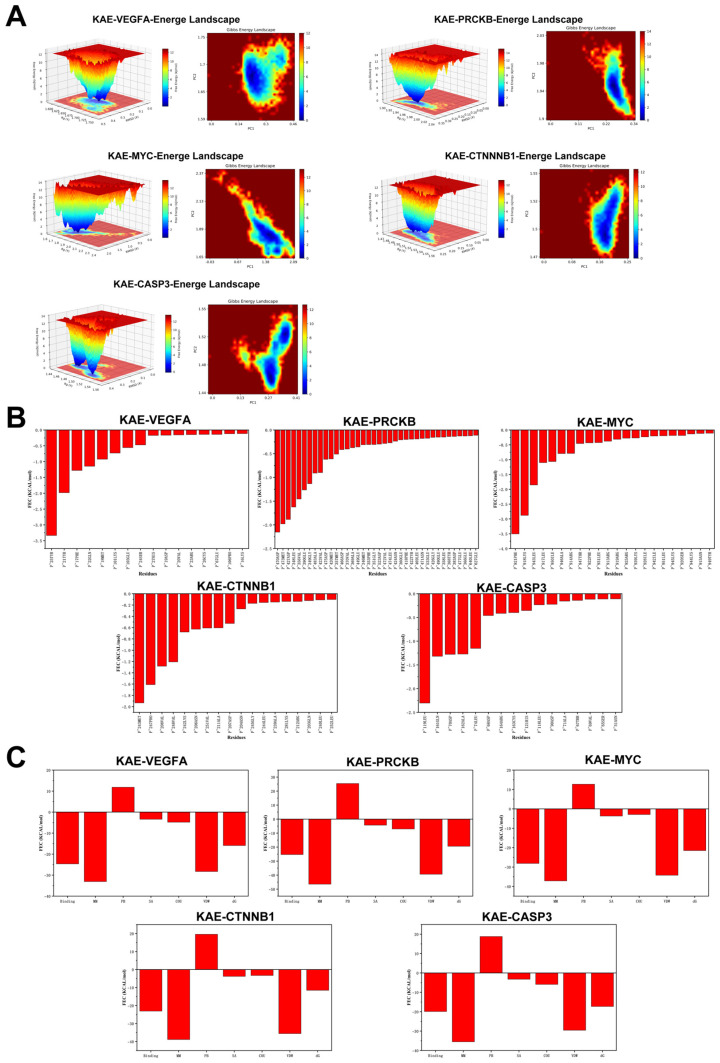
Free energy landscape analysis of kaempferol and core target complex. (**A**) Two-dimensional and three-dimensional mappings of the free energy landscape. (**B**,**C**) Residue energy decomposition for the binding of kaempferol to protein.

To rigorously evaluate kaempferol’s binding stability and conformational dynamics with the five hub targets, we performed 100 ns all-atom MD simulations under physiological conditions using GROMACS (2024.3). Trajectory analysis quantified RMSD, RMSF, intermolecular hydrogen bonds, Rg, and Gibbs free energy landscapes from PCA. RMSD analysis (Figure 3A) indicated that most complexes remained stable during simulation, although CASP3 showed some deviation after 50 ns, suggesting conformational drift. RMSF distribution (Figure 3B) revealed variable flexibility across regions, with all complexes showing relatively high overall flexibility. Rg analysis confirmed that most targets maintained compact, stable global folding (Figure 3C). SASA analysis (Figure 3D) showed that PRKCB had the highest solvent exposure (155–160 nm^2^), while MYC and CASP3 showed moderate exposure (78–82 nm^2^). Hydrogen bond analysis (Figure 3E) indicated that VEGFA, PRKCB, CTNNB1, and CASP3 maintained stable anchoring (maintaining an average of 1–4 hydrogen bonds throughout the 100 ns simulation), whereas MYC fluctuated more (Figure 4A). Gibbs free energy landscapes from the first two principal components showed that PRKCB and CTNNB1 had deep, focused energy wells (narrow 2D basins, sharp 3D valleys), indicating stable binding conformations near the global minimum. VEGFA and CASP3 showed moderate stability, while MYC displayed a more dispersed landscape (extended shallow basin), suggesting weaker stability. Per-residue free energy contribution (FEC) represents the contribution of protein residues to ligand binding (Figure 4B,C). In KAE-MYC, residue 921PHE reached −3.5 kcal/mol, and in KAE-VEGFA, residue 25TYR contributed >−3 kcal/mol, indicating that these residues make significant contributions to the protein–ligand interaction.

### 2.4. Effects of Kaempferol on AGE-BSA-Induced HK-2 Cell Viability and Oxidative Stress Markers

**Figure 5:**
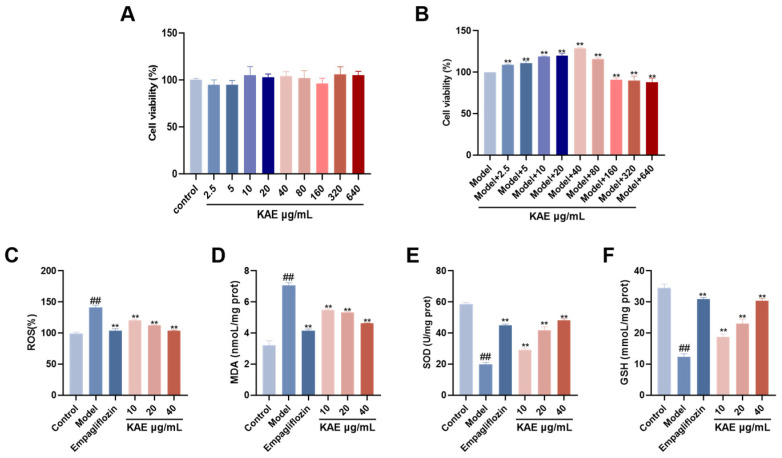
The effects of kaempferol on the viability and oxidative stress indicators of HK-2 cells induced by AGE-BSA (($\bar{x}$ ± s), *n* = 3). (**A**,**B**) Activity of HK-2 cells cultured with a concentration gradient of kaempferol. (**C**–**F**) Kaempferol inhibits the oxidative stress of HK-2 cells under DN pathology: (**C**) ROS levels, (**D**) MDA levels, (**E**) SOD levels, (**F**) GSH levels. Note: ## *p* < 0.01, vs. Control; ** *p* < 0.01, vs. Model.

To assess the cytotoxicity of kaempferol, HK-2 cells were treated with various concentrations (1.25–640 μg/mL) for 24 h. Kaempferol exhibited no obvious cytotoxicity across all tested concentrations (Figure 5A). The MTT assay (Figure 5B) revealed that kaempferol at 1.25–40 μg/mL increased cell viability in a concentration-dependent manner compared with the model group, with the maximal protective effect at 40 μg/mL, whereas viability decreased at concentrations ≥ 80 μg/mL. Accordingly, 40 μg/mL was selected as the high dose, with 10 μg/mL (low) and 20 μg/mL (medium) used for subsequent mechanistic studies.

Oxidative stress markers are shown in Figure 5C–F. Compared with the control group, the model group exhibited significantly elevated levels of ROS and MDA (*p* < 0.001), along with markedly reduced SOD activity and GSH level (*p* < 0.01). Conversely, kaempferol treatment groups showed significantly decreased ROS and MDA levels (*p* < 0.05) and increased SOD and GSH levels (*p* < 0.05) relative to the model group. These results indicate that kaempferol effectively alleviates AGE-BSA-induced cellular oxidative stress injury.

### 2.5. Effects of Kaempferol on AGE-BSA-Induced Expression of Fibrosis Markers in HK-2 Cells

**Figure 6:**
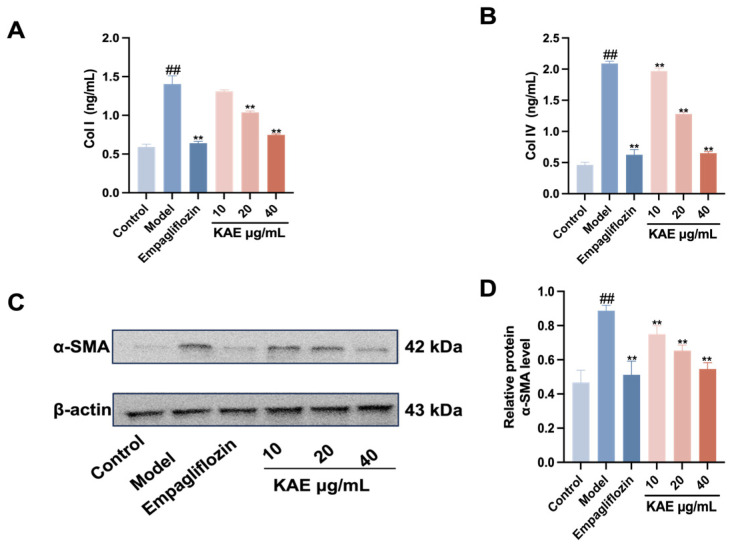
The effects of kaempferol on the fibrosis markers of HK-2 cells induced by AGE-BSA (($\bar{x}$ ± s), *n* = 3). (**A**) Col I levels. (**B**) Col IV levels. (**C**) Western blot analysis of α-SMA in each group. (**D**) α-SMA levels. Note: ## *p* < 0.01, vs. Control; ** *p* < 0.01, vs. Model.

To further evaluate renal fibrosis, the expression of α-SMA, Col I, and Col IV was determined (Figure 6). A marked upregulation of these fibrotic markers was observed in the model group compared with the control group (*p* < 0.05). Notably, kaempferol treatment at all tested doses significantly reversed this upregulation (*p* < 0.05). These results demonstrate that kaempferol suppresses the expression of fibrosis-related proteins, thus conferring multifaceted protection against diabetic nephropathy-induced injury in renal tubular epithelial cells.

### 2.6. Effect of Kaempferol on Inflammatory Cytokine Levels in HK-2 Cells

**Figure 7:**
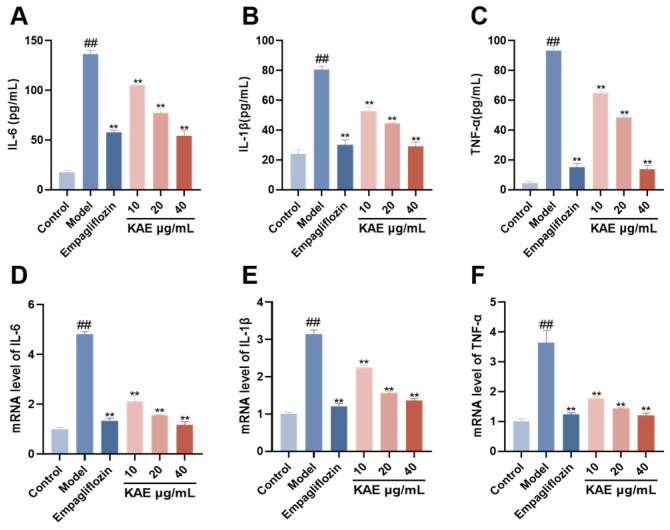
The ameliorative effect of kaempferol on the inflammatory response in HK-2 cells under diabetic nephropathy (DN) conditions (($\bar{x}$ ± s), *n* = 3). (**A**–**C**) Effects of kaempferol on the levels of inflammatory cytokines in HK-2 cells: (**A**) IL-6, (**B**) IL-1β, (**C**) TNF-α. (**D**–**F**) Effects of kaempferol on the mRNA expression of inflammatory factors in HK-2 cells under DN pathology: (**D**) IL-6, (**E**) IL-1β, (**F**) TNF-α. Note: ## *p* < 0.01, vs. Control; ** *p* < 0.01, vs. Model.

Inflammatory cytokine levels (Figure 7) showed, compared with the control group, that the model group had significantly higher levels of TNF-α, IL-6, and IL-1β at both protein and mRNA levels (*p* < 0.05). Conversely, kaempferol treatment significantly reduced these levels (*p* < 0.05). These results indicate that kaempferol exerts a protective effect on renal tubular epithelial cells in diabetic nephropathy by suppressing inflammatory cytokine expression.

### 2.7. Kaempferol Reduces RAGE Immunofluorescence in HK-2 Cells

**Figure 8:**
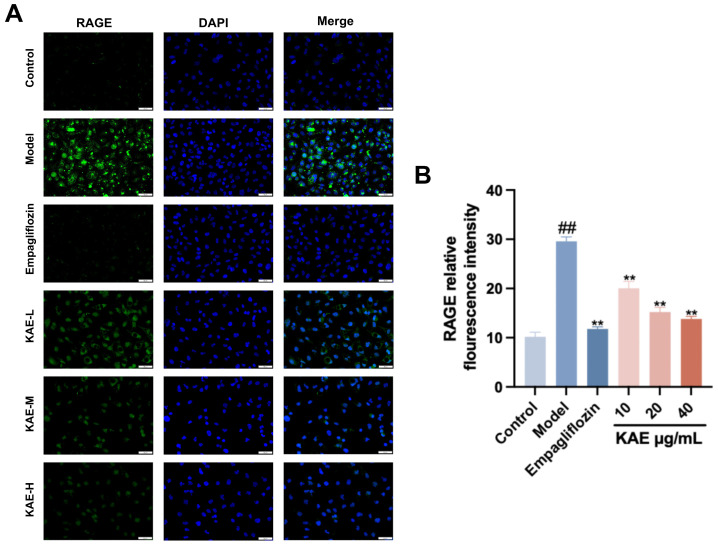
Effect of kaempferol on the expression of RAGE in HK-2 cells. (**A**) Immunofluorescence staining was used to detect the localization and expression of RAGE protein in HK-2 cells (scale = 50 μm); (**B**) quantitative analysis of the average fluorescence intensity of RAGE. Note: ## *p* < 0.01, vs. Control; ** *p* < 0.01, vs. Model.

Immunofluorescence (Figure 8) showed significantly higher RAGE fluorescence intensity in the model group versus control (*p* < 0.001). Compared to the model group, empagliflozin, all kaempferol doses, and RAGE blockade significantly reduced RAGE fluorescence (*p* < 0.001). These results indicate that kaempferol effectively inhibits AGE-BSA-induced RAGE expression in HK-2 cells, suggesting renal protection via RAGE signaling modulation.

### 2.8. Kaempferol Suppresses AGEs, RAGE, p-PKCβII, and TGF-β1 Expression

**Figure 9:**
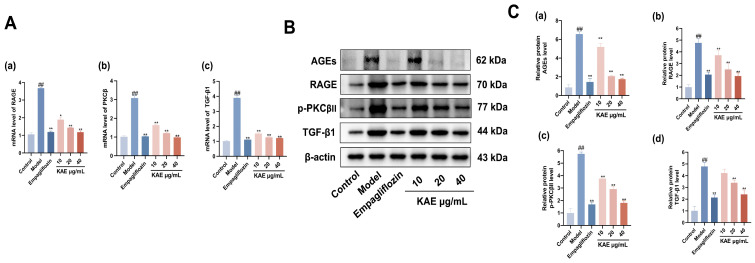
Assessment of the effect of kaempferol on alleviating DN via AGE/RAGE/PKCβ/TGF-β1 pathway mRNA and protein expression (($\bar{x}$ ± s), *n* = 3). (**A**) mRNA analysis: (**a**) RAGE levels. (**b**) PKCβ levels. (**c**) TGF-β1 levels. (**B**) Western blot analysis of RAGE, p-PKCβII, and TGF-β1 in each group. (**C**) Quantitative analysis of Western blot for (**a**) AGE levels, (**b**) RAGE levels, (**c**) p-PKCβII levels, (**d**) TGF-β1 levels. Note: ## *p* < 0.01, vs. Control; * *p* < 0.05, ** *p* < 0.01, vs. Model.

PCR analysis (Figure 9A) demonstrated that the mRNA levels of PKCβ, RAGE, and TGF-β1 in HK-2 cells were significantly higher in the model group than in the control group (*p* < 0.01). Conversely, treatment with kaempferol led to a significant reduction in the mRNA expression of the aforementioned genes compared with the model group (*p* < 0.05). Western blot (Figure 9B,C) revealed significantly elevated AGEs, p-PKCβII, RAGE, and TGF-β1 levels in model versus control cells. These abnormalities decreased dose-dependently with kaempferol. High-dose kaempferol strongly inhibited all three proteins (*p* < 0.01), comparable to empagliflozin. Medium-dose kaempferol significantly highly inhibited RAGE and TGF-β1 (*p* < 0.01), and significantly inhibited p-PKCβII (*p* < 0.01). Low-dose kaempferol significantly inhibited RAGE and p-PKCβII (*p* < 0.05) but did not significantly affect TGF-β1 (*p* > 0.05). These findings suggest that kaempferol protects renal tubular epithelial cells in DN by inhibiting the AGE/RAGE/PKCβ/TGF-β1 signaling pathway.

## 3. Discussion

DN has become a major medical challenge, with pathogenesis closely tied to chronic inflammation, immune dysfunction, and various complications [18]. Given DN’s complexity, multi-component TCMs can exert synergistic effects through multi-target, multi-pathway mechanisms. Astragalus root, widely used in TCM, has shown DN treatment potential in both clinical and experimental settings [19,20]. However, its complex composition means that the specific pharmacological mechanisms remain incompletely defined. This study therefore employed a comprehensive strategy integrating network pharmacology, molecular docking, molecular dynamics simulation, and in vitro validation to systematically analyze Astragalus root’s active components and their DN-related targets, with particular focus on kaempferol.

Through network pharmacology, we identified five key Astragalus targets against DN: CASP3, VEGFA, CTNNB1, MYC, and PRKCB. CASP3, a key apoptosis executor, is activated by high glucose in DN, mediating pyroptosis and worsening tubulointerstitial injury [21]. VEGFA, a core regulator of angiogenesis and vascular permeability, is abnormally upregulated in early DN and closely linked to proteinuria [22]. CTNNB1, a Wnt/β-catenin pathway component, maintains podocyte adhesion, differentiation, and survival; its dysregulation disrupts podocyte integrity and the glomerular filtration barrier [23]. MYC regulates proliferation and fibrosis; high glucose induces its expression, promoting profibrotic gene transcription [24]. PRKCB (PKCβ) is central to DN pathogenesis; high glucose activates PKC signaling, inducing glomerular basement membrane thickening, mesangial expansion, and ECM deposition [25]. These findings confirm that these targets are closely associated with DN progression and that Astragalus components can modulate them. Molecular docking showed that kaempferol had high binding affinity for all five targets. MD simulations further confirmed the stability of kaempferol–target complexes throughout the simulation, with small backbone fluctuations and sustained key hydrogen bonds, validated by both energetic and dynamic structural analyses.

GO enrichment revealed that Astragalus root’s DN mechanisms involve positive regulation of epithelial cell proliferation, secretory granule lumen, and protein serine/threonine kinase activity. KEGG analysis identified the AGE-RAGE pathway as critical. The AGE-RAGE pathway links hyperglycemia to renal injury through oxidative stress, inflammation, fibrosis, and cell death, making it an important DN treatment target [26]. Modulating AGE-RAGE signaling is an effective DN strategy. For example, isoliquiritigenin, a natural flavonoid, shows preventive DN potential by inhibiting this pathway [27]; Rehmannia–Cornus combinations alleviate DN-induced renal injury through AGE-RAGE regulation [28]; Niaoduqing Granules attenuate high glucose-induced AGE-RAGE activation and ACE overexpression in MPC5 cells [29]; and Yiqi Yangyin Tongluo Formula ameliorates DN in rats by inhibiting AGE-RAGE and reducing oxidative stress [30]. Our network pharmacology analysis similarly confirmed AGE-RAGE signaling as key to kaempferol’s renal protection.

Based on bioinformatics results, we established an in vitro DN cell model to validate kaempferol’s therapeutic potential, focusing on renal fibrosis, oxidative stress, and inflammation. Renal fibrosis and function loss are key DN features driving progression to end-stage disease. High glucose promotes myofibroblast transformation and excessive ECM deposition, leading to glomerulosclerosis and interstitial fibrosis [31]. α-SMA, Col I, and Col IV are key effector molecules in this process; their abnormal changes directly contribute to structural remodeling and functional decline [32]. Our results showed that kaempferol significantly reduced these fibrosis markers, indicating renal protection via reduced ECM deposition and fibrosis. Empagliflozin was selected as a positive control due to its proven efficacy in significantly delaying the decline in renal function and reducing proteinuria in patients with DN [33].

Oxidative stress and inflammation remain central to DN pathogenesis. Immune-mediated inflammation plays a critical role; innate immune cell activation enhances pro-inflammatory responses through autocrine and paracrine factors, amplifying inflammation and damaging the kidney [34]. TNF-α, IL-6, and IL-1β, as M1 macrophage markers, accelerate DN inflammation and serve as key biomarkers [35]. Persistent hyperglycemia activates NADPH oxidase, leading to excessive ROS production and reduced SOD and GSH, triggering oxidative stress [36]. Our study showed that kaempferol reversed DN-induced increases in TNF-α, IL-6, IL-1β, ROS, and MDA, as well as reduced GSH and SOD activity, indicating modulatory effects on oxidative stress and inflammation.

The AGE-RAGE-PKCβ-TGF-β1 axis is a core signaling bridge linking hyperglycemic metabolic disorders to renal structural remodeling [37]. High glucose increases AGE production; AGE accumulation and RAGE binding initiate pathological cascades, activating PKC, MAPK, and NF-κB pathways [38]. Activated PKCβ upregulates TGF-β1 expression [39]. TGF-β1 induces EMT and excessive ECM deposition, upregulating myofibroblast transformation (α-SMA) and ECM deposition (Col I, Col IV) through Smad-dependent and -independent pathways, reducing ECM degradation and increasing deposition, ultimately driving renal interstitial fibrosis and glomerulosclerosis [40,41,42]. This axis also intertwines with oxidative stress and inflammation, forming DN’s complex molecular network. Berberine reduces AGE production and inhibits AGE-RAGE-PKCβ-TGF-β1 overactivation to protect the kidneys [43]. Olmesartan downregulates the AGE/PKC/TGF-β1 axis, modulating oxidative stress, inflammation, fibrosis, and autophagy [44]. Fluofenidone protects renal function by inhibiting AGE-RAGE and PKC pathways, suppressing diabetes-induced renal oxidative stress [45]. Our Western blot results showed significantly elevated AGEs, RAGE, p-PKCβII, and TGF-β1 in AGE-BSA-induced HK-2 cells, with kaempferol effectively suppressing these abnormalities. Immunofluorescence confirmed that kaempferol reduced RAGE fluorescence intensity in model cells. Thus, kaempferol likely combats DN partly through AGE-RAGE-PKCβ-TGF-β1 pathway regulation.

Despite providing valuable insights, this study has limitations. First, our findings are primarily from cell models and lack clinical data; future studies should combine in vivo animal experiments and clinical validation to systematically evaluate the pharmacodynamics, histopathological changes, target protein expression, as well as the pharmacokinetics and safety of kaempferol. Second, AGE-RAGE interaction in DN activates multiple downstream pathways beyond PKC, including MAPK and NF-κB; future research will explore kaempferol’s effects on these additional pathways. Overcoming these limitations will enable more systematic understanding of kaempferol’s regulatory mechanisms in DN and facilitate clinical translation.

## 4. Materials and Methods

### 4.1. Reagents and Chemicals

Kaempferol (CAS No. 520-18-3) was obtained from Shanghai Standard-Tech Co., Ltd. (Shanghai, China). HK-2 cell culture medium (Cat. No. iCell-h096-001b) was purchased from Cyagen Biosciences (Shanghai) Co., Ltd. (Shanghai, China). Trypsin (Cat. No. BC-CE-005), PBS buffer (Cat. No. BC-BPBS-01), and fetal bovine serum (Cat. No. BC-SE-FBS01) were supplied by Nanjing Senbeijia Biotechnology Co., Ltd. (Nanjing, China). MTT (Cat. No. 1334GR001) was purchased from neoFroxx GmbH (Eningen, Germany). Empagliflozin (Cat. No. HY-15409) was obtained from MedChemExpress LLC (Monmouth Junction, NJ, USA). AGE-BSA (Cat. No. 22968) was purchased from Cayman Chemical Company (Ann Arbor, MI, USA). Membrane lysis buffer (Cat. No. G1204), DAPI staining reagent (Cat. No. G1012), and bovine serum albumin (BSA, Cat. No. GC305010) were purchased from Wuhan Servicebio Technology Co., Ltd. (Wuhan, China).

Primary antibodies were sourced as follows: anti-AGEs (Cat. No. bs-1158R, Bioss, Beijing, China); anti-RAGE (Cat. No. ab216329, Abcam, Cambridge, UK); anti-p-PKCβII (Cat. No. HA723796, Huabio, Hangzhou, China); anti-RAGE (Cat. No. ER64753, Huabio, Hangzhou, China); anti-TGF-β1 (Cat. No. 21898-1-AP, Proteintech, Rosemont, IL, USA); and anti-β-actin (Cat. No. AC026, Abclonal, Woburn, MA, USA). Secondary antibodies included goat anti-rabbit IgG (H+L) HRP (Cat. No. AS014, Abclonal, Woburn, MA, USA), HRP-conjugated goat anti-mouse IgG (H+L) (Cat. No. AS003, Abclonal, Woburn, MA, USA), and FITC-labeled goat anti-rabbit IgG (Cat. No. GB22303, Servicebio, Wuhan, China). Antibody dilutions were as follows: AGEs (Bioss) 1:2000; RAGE (Abcam) 1:300; p-PKCβII 1:250,000; RAGE (Huabio) 1:2000; TGF-β1 1:2000; β-actin 1:50,000; HRP-conjugated secondary antibodies 1:8000; and FITC-conjugated secondary antibody 1:100. ECL chemiluminescent substrate (ultra-sensitive, Cat. No. BL520B), protease inhibitor (Cat. No. BL612A), PMSF (Cat. No. BL507A), phosphatase inhibitor (Cat. No. BL615A), and BCA protein assay kit (Cat. No. BL521C) were all purchased from Biosharp (Hefei, China).

### 4.2. Network Pharmacology Analysis

#### 4.2.1. DN-Related Target Identification

To comprehensively collect DN-associated genes, we searched three databases—GeneCards (https://www.genecards.org, accessed on 7 January 2026), OMIM (https://omim.org, accessed on 7 January 2026), and TTD (https://db.idrblab.net/ttd, accessed on 7 January 2026)—using the keyword “diabetic nephropathy.” For GeneCards, a relevance score ≥ 1 was applied as the cutoff. Genes retrieved from each database were merged, and duplicate entries were removed to obtain a final set of DN-related targets.

#### 4.2.2. Astragalus Root Target Prediction

Active constituents of Astragalus root were screened from the TCMSP database (https://tcmsp-e.com, accessed on 3 January 2026) using criteria of oral bioavailability (OB) ≥ 30% and drug-likeness (DL) ≥ 0.18. To cross-validate and enrich the compound set, the Batman-TCM database (http://bionet.ncpsb.org.cn/batman-tcm/, accessed on 5 January 2026) was additionally searched with the same keywords, and duplicate compounds from the two databases were merged. Corresponding targets were then retrieved from the SwissTargetPrediction database (http://swisstargetprediction.ch, accessed on 5 January 2026. species: Homo sapiens; confidence threshold: 0.10). The overlap between Astragalus targets and DN-related targets was defined as the common genes mediating Astragalus root’s effects on DN.

#### 4.2.3. PPI Network Construction

Using the STRING database (https://string-db.org, accessed on 15 January 2026, version 11.5, species: Homo sapiens; confidence threshold: 0.40), we built a PPI network that included 882 DN-related genes and 381 Astragalus-DN target genes. TSV files were downloaded and analyzed with the CytoHubba plugin in Cytoscape (version 3.6.0) for network visualization and node centrality evaluation.

#### 4.2.4. Herb-Component–Target-Disease Network Construction

We constructed the herb-component–target-disease network by importing three datasets into Cytoscape (version 3.6.0): Astragalus drug targets, DN-related targets, and the intersecting genes from Section 4.2.2.

#### 4.2.5. Hub Gene Identification

To pinpoint core regulatory nodes, we systematically screened hub genes from the Astragalus-DN target set using the CytoHubba plugin in Cytoscape (version 3.6.0). Node centrality scores were calculated with the Maximum Clique Centrality (MCC) topological algorithm using density-weighted clustering. The top 10 candidate hub genes were ranked and extracted based on the scoring results.

#### 4.2.6. GO and KEGG Enrichment Analyses

To understand how Astragalus root acts against DN, we performed GO and KEGG enrichment analyses on the co-expressed genes. A significance threshold of *p* < 0.05 was applied, and the top-ranked terms were visualized as bubble charts and Sankey diagrams.

### 4.3. Molecular Docking

The crystal structures of the five previously identified core targets were retrieved from the Protein Data Bank (PDB), with a preference for high-resolution structures (typically <2.5 Å). Using PyMOL software (version 2.6.0), water molecules and irrelevant ions were removed. Missing amino acid residues in the active pocket were then filled using Modeller to construct complete binding pocket structures. Subsequently, hydrogen atoms were added and charges were assigned to the proteins to simulate the physiological environment.

For ligand preparation, the three-dimensional (3D) structures of the active components of Astragalus root were downloaded from the PubChem database. These structures were subjected to energy minimization using Chem3D software (version 24.0) to obtain stable low-energy conformations. Solvent molecules were removed with PyMOL, followed by hydrogenation and charge assignment using AutoDock Tools (version 1.5.6). The resulting key compounds were then docked against the five prepared protein targets. Those exhibiting favorable docking scores with all five targets were designated as the potential core components.

### 4.4. Molecular Dynamics Simulation

MD simulations were carried out to further validate binding interactions. Protein–ligand complex simulations were performed with the GROMACS 2024.3 package. The system was placed in a periodic boundary cubic box and solvated with the AMBER14SB force field and TIP3P water model. Small molecule topology files were generated using sobtop software, and NaCl was added to a final concentration of 150 mM to mimic physiological conditions. Following energy minimization and stepwise NPT equilibration under positional restraints (100 ps), a 100 ns production run was conducted with a 2 fs time step. Trajectories were post-processed to eliminate periodic boundary artifacts and center the system. Key stability metrics assessed included RMSD of protein backbone Cα atoms, Rg, RMSF, SASA, and the number of intermolecular hydrogen bonds between protein and ligand. Binding free energy (ΔGbind) was computed using the MM-PBSA method. To assess conformational stability, 2D and 3D free energy landscapes (FEL) were derived from PCA (PC1/PC2) to map the free energy distribution across conformational space. Dark blue low-energy regions represent global free energy minima, reflecting the most thermodynamically stable conformations.

### 4.5. Cell Culture

HK-2 cells in logarithmic growth phase were collected, washed with PBS, and trypsinized. After centrifugation at 250× *g* for 5 min, the supernatant was discarded and the pellet resuspended in fresh medium. Cell density was adjusted to 5 × 10^4^ cells/mL, and 100 μL of the suspension was seeded into each well of a 96-well plate (edge wells filled with sterile PBS). Cells were cultured at 37 °C in a 5% CO_2_ incubator. The DN model was established by exposing the cells to 200 μg/mL AGE-BSA for 24 h at 37 °C.

### 4.6. MTT Assay

After cell attachment, treatments were applied as described below, with four replicate wells per group.

Experiment 1 (drug toxicity screening): Control, kaempferol (1.25, 2.5, 5, 10, 20, 40, 80, 160, 320, 640 μg/mL); corresponding drugs were added and incubated for 24 h.

Experiment 2 (protective effect assessment): Control and model (AGE-BSA) + kaempferol (1.25, 2.5, 5, 10, 20, 40, 80, 160, 320, 640 μg/mL) groups. AGE-BSA was first added for 24 h to establish the model, followed by kaempferol treatment for 24 h.

After drug treatment, the supernatant was removed, and 200 μL of MTT solution (final concentration 0.5 mg/mL) was added to each well. The plate was gently shaken and incubated for an additional 1 h at 37 °C. The supernatant was carefully aspirated, and 150 μL of DMSO was added per well. The plate was shaken at low speed in the dark for 10 min to dissolve formazan crystals. Absorbance was measured at 570 nm using a microplate reader.

### 4.7. Measurement of Pharmacodynamic Evaluation Indicators

HK-2 cells were seeded into 6-well plates and then incubated with 200 μg/mL AGE-BSA, together with low, medium, and high concentrations of the active components. The levels of Col I, Col IV, oxidative stress markers (MDA, SOD, ROS, GSH), and inflammatory cytokines (TNF-α, IL-6, IL-1β) in each group were determined according to the manufacturer’s instructions of the respective assay kits.

### 4.8. Immunofluorescence

Cell slides were washed three times with PBS (5 min each). Cells were permeabilized with membrane lysis buffer for 10 min at room temperature, blocked with BSA for 20 min at room temperature, and then incubated overnight at 4 °C with anti-RAGE primary antibody (1:300). After three PBS washes, slides were incubated with FITC-labeled secondary antibody (1:100) for 30 min at 37 °C, followed by three PBS washes. DAPI staining was performed for 10 min at room temperature in the dark, followed by three final PBS washes. Images were captured, and mean fluorescence intensity was quantified using Image-J.

### 4.9. RT-qPCR Analysis

Total RNA was extracted from HK-2 cells using TRIzol, quantified by NanoDrop, and reverse-transcribed into cDNA with PrimeScript RT Master Mix. RT-qPCR was conducted using TB Green Premix Ex Taq II on a qTOWER2.0 system, with GAPDH as internal control, and data analyzed by the 2^−ΔΔCt^ method. Primer sequences are shown in Table 1.

### 4.10. Western Blotting

Total protein was extracted from HK-2 cells using RIPA buffer containing 1% protease inhibitor. Protein concentration was determined with a BCA kit (Elabscience, Wuhan, China). Samples were separated by 4–12% SDS-PAGE and transferred to PVDF membranes. After blocking with 5% non-fat milk for 1 h, membranes were incubated overnight at 4 °C with primary antibodies against AGEs (1:2000), p-PKCβII (1:25,000), RAGE (1:2000), TGF-β1 (1:2000), α-SMA (1:2000) and β-actin (1:50,000). Following three TBST washes, HRP-conjugated secondary antibodies (1:10,000) were added for 1 h. Bands were visualized by ECL. Statistical analysis was performed using *t*-tests, with *p* < 0.05 considered significant.

### 4.11. Statistical Analysis

Data were processed with SPSS 26.0 and GraphPad Prism 8.5.0. Normality of the measurement data was assessed using the Kolmogorov–Smirnov and Shapiro–Wilk tests. Data conforming to a normal distribution were analyzed using one-way analysis of variance (ANOVA). In cases of homogeneity of variance, multiple comparisons were performed using the least significant difference (LSD) *t*-test; in cases of heterogeneity of variance, the Games–Howell method was used for multiple comparisons. For measurement data that violated the assumption of homogeneity of variance, intergroup comparisons were performed using the Mann–Whitney U test. Results are expressed as mean ± SD, with *p* < 0.05 indicating statistical significance.

## 5. Conclusions

**Figure 10:**
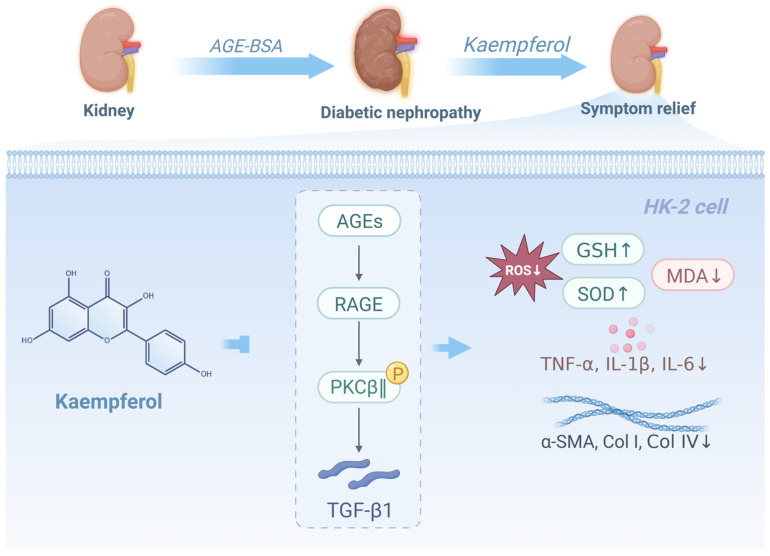
Mechanisms of kaempferol in improving DN.

In summary, this study demonstrates that kaempferol, a key bioactive constituent of Astragalus root, exerts renoprotective effects against diabetic nephropathy (DN) through modulation of the AGE-RAGE-PKCβ-TGF-β1 signaling pathway. This multi-target regulatory mechanism involves inhibition of ECM deposition, oxidative stress, inflammation, and renal fibrosis, which aligns well with the complex pathophysiology of DN (Figure 10).

Compared with conventional agents that primarily target single pathways, kaempferol offers a promising complementary strategy. Although further in vivo and clinical studies are needed to confirm efficacy and safety, our findings provide a scientific basis for the clinical application of Astragalus root, and its active constituent kaempferol, in DN management.

## Figures and Tables

**Table 1:** Primers sequences used for RT-qPCR.

Genes	Forward Primer (5′-3′)	Reverse Primer (5′-3′)
IL-6	CACAAGTCCGGAGAGGAGAC	ACAGTGCATCATCGCTGTTC
IL-1β	AGGCTGACAGACCCCAAAAG	CTCCACGGGCAAGACATAGG
TNF-α	ATGGCCTCCCTCTCATCAGT	TTTGCTACGACGTGGGCTAC
PKCβ	AGCCCCACGTTTTGTGACC	GCTGGGAACATTCATCACGC
RAGE	AGGTGAGTGGAGAAAGCCAG	ATGTGTCAGGTGTTTAATCA
TGF-β1	CTGCTGACCCCCACTGATAC	GGCTGATCCCGTTGATTTC
β-actin	ACCTCTATGCCAACACAGTG	GGACTCATCGTACTCCTGCT

## Data Availability

The original contributions presented in this study are included in the article. Further inquiries can be directed to the corresponding authors.

## Abbreviations

The following abbreviations are used in this manuscript:

BP	Biological Process
CC	Cellular Component
KAE	Kaempferol
GSH	Glutathione
IL-1β	Interleukin-1β
IL-6	Interleukin-6
MCC	Maximum Cluster Centrality
MD	Molecular Dynamics
MDA	Malondialdehyde
MF	Molecular Function
Rg	Radius of Gyration
RMSD	Root-Mean-Square Deviation
RMSF	Root-Mean-Square Fluctuation
ROS	Reactive Oxygen Species
SASA	Solvent Accessible Surface Area
TCM	Traditional Chinese Medicine
DN	Diabetic Nephropathy
SOD	Superoxide Dismutase
ECM	Extracellular Matrix
Col I	Collagen Type I
Col IV	Collagen Type IV
FEL	Free Energy Landscapes
PPI	Protein–Protein Interaction
